# Parenting in the Context of Children’s Chronic Pain: Balancing Care and Burden

**DOI:** 10.3390/children5120161

**Published:** 2018-11-27

**Authors:** Jessica W. Guite, Beth S. Russell, Kendra J. Homan, Rebecca M. Tepe, Sara E. Williams

**Affiliations:** 1 Connecticut Children’s Medical Center, Hartford, CT 06106, USA; 2 Department of Pediatrics, University of Connecticut School of Medicine, Farmington, CT 06030, USA; 3 Department of Human Development and Family Studies, The University of Connecticut, Storrs, CT 06269, USA; beth.russell@uconn.edu; 4 Division of Behavioral Medicine and Clinical Psychology, Cincinnati Children’s Hospital Medical Center, Cincinnati, OH 45229, USA; Kendra.Homan@cchmc.org (K.J.H.); Sara.Williams@cchmc.org (S.E.W.); 5 Department of Pediatrics, University of Cincinnati College of Medicine, Cincinnati, OH 45267, USA; 6 Division of Social Services, Cincinnati Children’s Hospital and Medical Center, Cincinnati, OH 45229, USA; Rebecca.Tepe@cchmc.org

**Keywords:** children, parents, family, chronic pain, barriers to care, resiliency, human development, intervention

## Abstract

Parents of youth with chronic health conditions encounter numerous challenges in supporting their children across pediatric treatment contexts. Structural barriers to care, such as access issues and coordinating care across school, health, and family settings, can exacerbate challenges to daily functioning. Parents are often concomitantly managing their child’s chronic condition, their own health care needs, work and family demands. For these parents, accomplishing a manageable “work-life balance” feels elusive, if not impossible, when a chronic health condition is part of family life. Based on a recent symposium presentation, combined perspectives from the disciplines of pediatric psychology, parenting, and human development and family studies consider key challenges and opportunities to assist parent coping with stress associated with caregiving amidst pervasive changes in healthcare service delivery. Two innovative interventions to support parents in both an outpatient (“Parents as Coping Coaches”) and an inpatient (“Putting Parents FIRST”) context are described, with commonalities and unique aspects highlighted for each. These programs are considered in reference to a rapidly changing healthcare landscape, growing focus on the family as a core context for care, and importance of parent/caregiver self-care and crucial role in supporting children’s long-term health and resiliency.

## 1. Introduction

The need to support parents in the context of caring for a child with chronic pain is gaining more recognition as a cornerstone of effective treatment. This paper expands upon a recent symposium presented at the American Psychological Association Annual Convention focusing on parenting a child with chronic health challenges [1]. We begin by providing an overview of the family as a context for care and the important role of family dynamics and parenting behavior as it relates to youth with chronic pain. With this foundational background, we next consider specific parenting factors contributing to caregiving burden for parents of children with chronic pain conditions. Important aspects of interventions that support parents in caring for children with chronic conditions are described for interventions conducted in both outpatient and inpatient settings. 

### 1.1. Families: Context for Care

Interventionists face a need to develop theoretically grounded approaches to promote individual patient and family resilience to chronic pain within an ecologically valid and developmentally appropriate context of care. For families with a child or adolescent with a chronic pain diagnosis, the routines and relationships of the home are the context of care through which the family and its members may develop skills that promote adaptive responses to a diagnosis and its associated treatment. Family Systems theories describe families as a fully interactive collection of subsystems, where changes in one subsystem influence changes in all others [2,3]. For example, changes in parents’ well-being resulting from caregiving stress may influence parent–child interactions, thus shaping outcomes for the child, parent, and family overall. Interactions between family members can become patterned, thus forming dynamics that characterize how the family functions. Family dynamics combine to constitute parenting style, or parents’ consistent or patterned behavior used to interact with their child(ren) [4,5,6]. 

### 1.2. Parenting: Behavior and Styles

Like all behavior, parenting behavior often reflects both intrinsic and extrinsic influences (e.g., internal working models of the self and social world, and cultural and generational traditions/practices) [7,8,9,10]. As a result, parents’ behavior is shaped by sociocultural expectations that are sometimes left unspoken [11,12,13,14,15], yielding instances where parents respond to their children procedurally (i.e., relying on parenting behavior that may or may not be considered, examined, and enacted with coherent intent to manage the moment at hand). All parents draw from how they were parented, how they have seen others parent, and how they themselves have cared for others [10]. Expectations for how to parent that are set through past experiences are challenged when previous parenting prior to a child’s chronic pain diagnosis is no longer generalizable to the present, and when parents lack models for parenting in similar contexts (e.g., a peer group of parents facing similar challenges). 

Baumrind’s [5,16,17] influential description of parenting styles described a continuum of parenting behaviors on dimensions of warmth and control, and yielded three widely used typologies: (1) Authoritarian parenting: High in control, low in warmth, and characterized by parent demands with little opportunity for child input. (2) Authoritative parenting: Balanced on both dimensions, and characterized by demands that invite child input, often scaffolding child success for meeting parents’ expectations. (3) Permissive parenting: Low in control, high in warmth, characterized by little structure and few parental demands. More recent parenting typologies that have since emerged include Uninvolved/neglectful parenting (low in both dimensions) and Directive/protective parenting (high in both warmth and control dimensions) [18,19,20,21].

The study of parenting styles suggests there may be particular relationship dynamics that are amenable to intervention that promote positive individual and family outcomes. For example, improving parent-child communication and parents’ developmentally appropriate expectations can increase supportive parenting and reduce conflict or other harsh parenting behaviors associated with authoritarian and/or permissive parenting (styles characterized by intrusive or controlling behaviors, and by lax or disengaged behaviors, respectively). Both these typologies are associated with significant negative impacts on child development from infancy through adolescence [2,18,22,23]. Conversely, authoritative parenting (a style of parenting characterized by supportive interactions for promoting independent problem-solving) leads to children’s positive mental health outcomes and developmentally appropriate individuation throughout adolescence [18,22].

Questions remain about the characteristics of parenting styles with specific relevance for the study of families facing chronic pain. For example, parents’ own distress and the associated caregiving burden they feel pose risks to adaptive parenting behavior by taxing parents’ psychological resources (i.e., attention, patience, and supportive/warm emotional tenor that supports adaptive parent-child communication), especially in families that have a child with a chronic pain condition and face increased stressors compared to those with fewer enduring health concerns [24,25]. Parents’ emotional experiences—even of life-expectant stresses—vary, as do implications for individual/family wellbeing. Parents who practice adaptive self-care are better able to endure chronic stress with fewer negative individual outcomes and remain more emotionally available to attend and respond to their families in developmentally appropriate ways [26,27,28,29]. 

Families coping with pediatric chronic illnesses are characterized by more negative family functioning dynamics than families with healthy children, reporting parenting styles that are especially unresponsive and demanding at the same time [30,31,32,33]. Specifically, these families reportparenting styles that include less warmth and responsiveness, more control, and more overprotective behaviors. Observations of maternal overprotection, over-involvement, and enmeshment are common in families of patients with pain conditions [33,34,35]. Parenting styles characterized in this way are similarly described as anxious and identified as significant contributors to adolescent outcomes [36,37]. Parents who successfully modulate their own anxiety and distress through effective self-care are better poised to care for a child with a chronic pain condition. Parents of youth with chronic pain are perhaps more likely to use a Protective/Directive style of parenting, that reflects high warmth coupled with high control via explicit management, in response to their child’s distress and difficulty with managing their pain symptoms. The resulting parenting behaviors and parent-child interactions may appear to be more consistent with an earlier developmental stage (i.e., including more scaffolding, directive behavior, and control) as a protective response to guide their child through often demanding treatment plan components/routines. 

These emerging patterns in parenting observations in families coping with chronic pain may beshaped by parents’ emotional distress. Indeed, enduring stress and patterns of maladaptive coping create well-documented risks to individual and family functioning [38,39,40,41]. Further, adjustment to diagnosis, including catastrophizing and anxious parenting reactions, are salient in pediatric settings [42,43,44,45]. Interventions that address parents’ emotional wellbeing and coping skills may see additional benefits beyond the direct, parent-level impacts. Parents serve as the primary models of coping for their children, including modeling coping skills for their ill child. In the context of parenting a child with chronic pain, it is important for parents to learn to modulate their own distress around their child’s pain to be able to assist their child to effectively self-manage their pain. The emerging literature on these families often grounds research approach and study design in theories that emphasize social influences on individual behavior (e.g., social learning theory), given the cognitive process of learning through observation and parents’ crucial role as a primary source of socialization who model responses to challenging situations [46,47]. In addition, a compelling conceptual framework has recently emerged related to “chronic pain contagion” among families coping with pediatric chronic pain, that ongoing suffering is jointly experienced by the child and the empathic parent observer, producing maladaptive changes for both [48]. In the sections that follow, we will focus on parenting a child with chronic pediatric pain and the need for parent support across outpatient and inpatient treatment settings. 

### 1.3. Supporting Parent Resilience in the Context of Outpatient Pediatric Pain Clinic Treatment 

Pediatric chronic illness essentially creates added stress on the parent and family. This is an additional challenge to the typical parenting role, which naturally presents occasional stressors throughout development. The resulting caregiving burden poses risks to family functioning and parent health [49]. While many families do well, examples of common challenges include: (a) Finding appropriate levels of protective behaviors, control, and parental warmth and (b) building positive, developmentally appropriate relationships between parents and children [33]. 

Parenting youth with chronic pain specifically presents challenges that reflect the fact that this condition is common, costly, and associated with significant caregiving burden [50,51]. Pediatric chronic pain is common, with community samples of children/adolescents documenting that upwards of 83% had pain within a three month period and 30.8% had pain present for >6 months [52]. Among pediatric pain samples, the most common problem locations include headache (60.5%), abdominal (43.3%), limb (33.6%), and back pain (30.2%). A recent systematic review [53] documents wide prevalence rates for pain locations, with consistently the most common sites including headache, abdominal pain, musculoskeletal pain, multiple pains, back pain, listed in order of most to least prevalent. We know that chronic pediatric pain is more prevalent in females than males, pain prevalence increases with age, and higher pain prevalence is associated with psychosocial factors and lower socioeconomic status. Most concerning is evidence to support that this complex problem is increasing. Reports suggest that between 2004 and 2010 there was an 831% increase in hospital admissions for pediatric chronic pain [54]. This trend is further compounded by the significant economic costs of pediatric chronic pain. Among a recent sample of adolescents seeking interdisciplinary treatment for chronic pain in 2014, the total cost to society for adolescents with “moderate to severe” chronic pain was extrapolated to $19.5 billion annually [55]. The authors elaborate that the primary driver of these costs included direct medical costs, followed by productivity losses. Notably, it is parents who shoulder the primary burden for these factors when parenting a child with chronic pain. 

### 1.4. Parent Factors Identified within the Pediatric Pain Literature

Among treatment seeking families of youth with chronic pain, recent literature has identified that 50% of parents of youth with chronic pain also report having chronic pain themselves [56]. In addition to potential increased risk for chronic pain conferred via genetic factors, a parent’s personal experience of coping with their own chronic pain can have powerful implications for the support that they are able to provide their child to effectively self-manage their own pain. Recent literature supports that parents commonly hold beliefs about their child’s pain that are associated with worry and heightened parenting stress [57]. Parental pain catastrophizing (i.e., parents’ catastrophic thinking about their child’s pain) [58], has been found to significantly contribute to parenting stress, parental depression and anxiety, as well as the child’s disability and school attendance above and beyond the child’s own experience of pain. Parent-adolescent relationships in families coping with pediatric chronic pain on the whole show less adolescent-parent relationship distress when compared to healthy adolescent-parent normative data [59]. Within this sample, we further found that adolescents reporting greater pain severity had parents who reported less adolescent-parent relationship distress and the relationship between adolescent’s pain severity and functional disability were more closely linked among dyads reporting less adolescent-parent relationship distress [59]. 

Parent pain catastrophizing, as well as parental protective responses to their child’s pain [60], have both been shown to contribute to complex, bi-directional patterns of dyadic interactions between parents and their child with chronic pain. For example, prior research supports that pain catastrophizing serves as a mediator of relationships between: (1) Pain and disability and (2) protective parenting responses and disability for adolescents with musculoskeletal pain [61] and that these relationships persist over time [62]. The latter study showed that parental protectiveness was associated with disability indirectly through pain catastrophizing at an initial pain clinic visit and follow-up [62], which collectively suggest efforts to modify both adolescent catastrophic pain beliefs and parental responses to pain are likely to improve adolescent functioning. The emerging parenting style of Protective/Directive parenting (high in both warmth and control dimensions) [19,20] holds promise for better understanding these relationships as previously noted. 

### 1.5. Pediatric Pain Treatment

The treatment of pediatric chronic pain is fundamentally interdisciplinary in nature [63,64]. Currently there is good support for behaviorally based interventions for pediatric chronic pain which are typically anchored in cognitive behavioral therapy (CBT) and acceptance and commitment therapy (ACT) treatment models [65,66]. The growing focus on including parents directly in treatment for youth with common chronic medical conditions (including chronic pain) has shown positive effects primarily for parent behavior post treatment, but less impact on child outcomes to date [67]. Despite this, few studies have published content of parent intervention or examined the feasibility or efficacy of implementing such programs within pediatric treatment contexts. Our discussion will now shift focus to consider two recently developed parent-focused interventions for youth with chronic pain. The first describes a newly developed outpatient intervention for parents of youth with chronic pain, “Parents as Coping Coaches,” while the second intervention was developed to be delivered in the context of intensive inpatient rehabilitation, “Putting Parents FIRST.” 

## 2. Methods, Measures and Results

### 2.1. Outpatient Treatment Context: The “Parents as Coping Coaches (PaCC)” Intervention

Parents as Coping Coaches (PaCC) [45] is an intervention designed to support the needs of parents of outpatient adolescents with chronic pain and initially created in 2015 as a research opportunity to address unmet clinical needs in a NE interdisciplinary pain clinic. Goals were to support parents to manage their own distress parenting a child with chronic pain with the goal of increasing distress tolerance (DT) and resilience and decreasing parent caregiving burden. We also sought to explore parental DT and child outcomes (e.g., reduced adolescent functional disability). The intervention was designed to be a brief, group, parent-only format that was intended to balance parent’s time away from their family with parent-focused time and providing a feeling of community. Parents attend three, 120 min, consecutive weekly sessions (3–6 participants per group). Group content covered three primary domains: (1) Pain Education: Introduced gate control theory of pain and misguided helping; (2) Parent-Adolescent Communication: Focused on joint problem solving and promoting developmentally appropriate levels of autonomy; and (3) Coping Skills: Focused on distress tolerance skill building and self-care. Daily diaries were also utilized and encouraged parent practice of strategies on days between group meetings. 

### 2.2. Parents as Coping Coaches (PaCC): Initial Feasibility and Acceptability

The group protocol was initially examined with a sample of parents and adolescents (*N* = 22) with idiopathic chronic pain recruited from an outpatient pediatric pain clinic of a children’s hospital in the Northeast, USA. Parents (of adolescents between the age 12 and 18 years (*M* = 15.2; 68% female) consented to participate in this Institutional Review Board (IRB) approved protocol (#15-088). The following self-report, pre-post treatment measures were included in the project: The Bath Adolescent Pain—Parental Impact Questionnaire (BAP-PIQ) [68] as a measure of caregiver burden which included eight subscales; The Distress Tolerance Scale (DTS) [69] utilizing the total score and four subscales: Tolerance of emotional distress, Appraisal of distress, Absorption—attention being absorbed by negative emotions, and Regulation—efforts to relieve distress; The Adult Responses to Children’s Symptoms (ARCS) [60] and utilized the Protect, Solicitousness, Monitor, Minimize, Distract subscales; and the Functional Disability Inventory (FDI) [70]. Acceptability of PaCC was assessed using a 20 item “Parent Feedback Survey,” that measured treatment satisfaction, acceptability of the intervention, and elicited suggestions to improve the experience for future groups. 

Results of the pilot study demonstrated good feasibility and acceptability for PaCC and have been previously documented in full detail [45]. In summary, 63% participants consented at clinic received the intervention and the remaining *N* = 13 (37%) who withdrew prior to group start indicated challenges common to participation in any type of group-based treatment [e.g., “Time/schedule” (*N* = 8), “Distance/ transportation” (*N* = 4), and “Pursuing other treatment” (*N* = 1)]. Most parents enrolled attended all sessions (68%), provided follow up data (95%), and were “very satisfied” with the three weekly sessions based on responses to the Parent Feedback Survey. Participation in the PaCC intervention was related to decreases in caregiving burden, protective and monitoring parenting responses to the adolescent’s pain, and parent-perceived adolescent pain burden and disability. Specifically, pre-post intervention change was assessed using paired samples t-tests with effect sizes reported for all study measures [45]. Most notable were medium to large effect sizes and significant post-test effects for decreased caregiving burden were found on the BAP-PIQ Total Score [t(16) = 3.67, *p* < 0.002] and the following subscales: Depression [t(20) = 2.53, *p* < 0.05], Anxiety [t(20) = 3.13, *p* < 0.01], Self-Blame/Helpfulness [t(20) = 3.15, *p* < 0.05], and Parental Behavior [t(20) = 4.86, *p* < 0.001] and for protective parenting responses to the adolescent’s pain on the following ARCS subscales: Protect [t(20) = 2.27, *p* < 0.05] and Monitor [t(20) = 5.46, *p* < 0.001]. Parent reports of adolescent functional disability showed and medium effect size and decrease from pre- to post-intervention [t(20) = 2.82, *p* < 0.05]. Feasibility, acceptability and promising preliminary efficacy was shown for PaCC, which provided parents with peer support along with pain education, communication and emotion regulation skills to alleviate caregiving burden. Future research will examine the extent to which PaCC may foster a parenting environment that encourages effective adolescent pain self-management. While these findings hold promise for PaCC, the target population is parents of youth treated in an outpatient care setting. This intervention was not specifically designed for families with greater impairment, such as those requiring more intensive inpatient pediatric pain treatment, which will be considered in the next section. 

### 2.3. Inpatient Treatment Context: The “Putting Parent FIRST” Intervention

Despite receiving adequate outpatient intervention, a subset of pediatric patients with chronic pain struggle to adhere to make functional improvement and require a higher level of care [71,72,73]. These patients require an intensive inpatient multidisciplinary pain management program where the complex interplay of the biological, psychological, and sociocultural factors of chronic pain are addressed simultaneously [74,75]. Similar to the home environment and in outpatient levels of care, parents play an important role within intensive multidisciplinary pain management programs [67]. Typically, intensive multidisciplinary pain management programs promote helping patients regain independence by taking a self-management approach to pain [67,74,75]. The patient’s pain experience and learned ability to cope, however, is influenced by those around them, particularly their parents/caregivers [76]. As a result, it can be challenging to identify parents’ role in treatment while balancing functional independence and accountability. Two recent studies have demonstrated that parent behavior changes positively relates to children’s pain and function outcomes when parents are included in children’s interdisciplinary inpatient pain treatment [77,78]. In this section, we describe the creation of a parent education program delivered in the context of an inpatient pain rehabilitation program, the Functional Independence Restoration (FIRST) program, and examine parents’ responses to the implementation.

### 2.4. Putting Parents FIRST: Development and Initial Feasibility and Acceptability Findings

Putting Parents FIRST is an intervention designed to educate parents on chronic pain, effective parenting methods, and behavior management techniques to improve their child’s long-term functional outcomes. Parents participated in weekly one-hour parent education group sessions as part of their child’s treatment in our intensive inpatient pediatric pain rehabilitation program. There are few published assessments of parent specific education programs within pediatric pain rehabilitation programs [78]. On average, group size was 2.5 parents per session. Each session consisted 30–45 min of educational material followed by 15–30 min of group discussion on applying the information learned during the session (totaling 60 min weekly). Sessions were co-led by a psychologist and social worker. The psychoeducational groups covered three primary domains. The first session focused on pain education to introduce parents to the neurological understanding of pain mechanisms in the brain (e.g., gate control theory of pain), the chronic pain and disability cycle, and the concept of “miscarried helping,” where support with the best of intentions backfires to perpetuate cycles of pain and disability (e.g., instructing parents not to ask about pain and place attention on function instead). The second session focused on parenting skills, including a self-assessment of parenting styles and introduction to authoritative parenting practices, with a specific focus on how to apply this type of parenting to children with chronic pain, such as setting expectations for normal function. The third session focused on transitioning home from intensive inpatient rehabilitation with an introduction to behavior management (e.g., writing a behavior contract, delineating parent/child expectations for following a schedule, and consequences for not doing so) and positive communication practices regarding functional expectations at home. 

Immediately following each session, parents completed a five-item measure asking them to evaluate acceptability of the session content through answering the following items: (1) This education session was useful/relevant; (2) This education session was easy to understand; (3) I liked this educational session; (4) I learned something new in the educational session; and (5) Based on the information covered in today’s session, I plan to make changes in how I handle my child’s pain. Parents rated their evaluations on a five-point Likert scale (0–4) from *strongly disagree* (0) to *strongly agree* (4). Scores were summed by item and mean total scores were calculated to obtain an overall composite score. Higher scores reflected more positive evaluation of the program.

The program was implemented in March 2018 and data were collected through October 2018. In this time, 25 caregivers from 19 families participated in the sessions. Examination of parents’ responses to education sessions was approved by the hospital’s Institutional Review Board (protocol 2015-8104). Among those were 17 mothers, seven fathers, and one grandmother. There were 20 responses from session one (pain education), 24 responses from session two (parenting skills), and 11 responses from session three (transitioning home). All parents were white and non-Hispanic (10% of children were bi-racial), and their children ranged in age from 9 to 18 years old (*M* = 15.32, *SD* = 2.58). 

First, each item was examined descriptively. The composite mean for all sessions was 3.73 (*SD* = 0.35); by session, the pain education mean was 3.79 (*SD* = 0.29), the parenting mean was 3.69 (*SD* = 0.35), and the transition mean was 3.71 (*SD* = 0.42). 

Next, univariate analyses of variance (ANOVAs) were conducted for the overall composite score and each of the five item scores between education sessions. There were no significant differences between sessions for the overall composite score or any individual item scores. Overall, all items were rated highly by parents in each session with average values ranging from 3.69/4.0 to 3.79/4.0. Thus, these initial feasibility and acceptability findings provide initial support for the Putting Parents FIRST intervention, which provided parents with targeted intervention while their children simultaneously participated in an inpatient chronic pain rehabilitation program. Future research will examine the extent to which Putting Parents FIRST may relate to children’s pain and function outcomes, as well as the potential effect on parent distress or adoption of a more adaptive parenting style. These findings are limited to the experience of parents of children with high levels of pain and disability related to chronic pain. 

## 3. Discussion

There is growing evidence that parents of children with chronic pain play a significant role in their children’s pain experience and as such, it is critical that parents play as active a role in their child’s treatment [77]. It can be challenging to fully appreciate a parent’s role in their child’s treatment in both outpatient and inpatient contexts. As examined in this paper, emerging intervention development research suggests that supporting parents in both outpatient and inpatient settings is both feasible and beneficial. Two newly created parent-focused interventions for parents of youth with chronic pain were considered in closer detail. Specifically, “Parents as Coping Coaches (PaCC),” a three-session outpatient group treatment for parents of youth was highlighted and shows positive effects for alleviation of caregiving burden [45]. Similarly, findings for parents of youth participating in “Putting Parents FIRST,” also a three-session group intervention delivered in an inpatient setting to parents of youth with chronic pain shows promise. Specifically, preliminary feasibility and acceptability findings indicated that parents participating in education sessions during their child’s inpatient pain treatment rated the sessions as highly useful, educational, enjoyable, clear, and impactful on their behavior toward their child. Further development and assessment of these newly created programs are underway. 

These new parent-focused interventions join the ranks of a growing number of behaviorally based treatment resources emerging for families challenged with chronic pediatric pain [79,80,81]. Specifically, we note that the existing emerging interventions to support the needs of parents of youth with chronic pain vary from intensive one-day workshops [79] to 6–8 weeks commitments [82,83]. PaCC’s three-session format was selected purposefully in order to balance challenges expressed by busy parents to limit the amount of time spent away from family responsibilities, while also maximizing the opportunity for parents to develop a sense of community by repeated contact with other parents struggling with similar issues. Putting Parents FIRST had a three-session format, selected purposefully to coincide with once weekly family days that were already scheduled in the program. Patients’ length of stay varies depending on need (average: three weeks). As a result, we sought to create material that could be delivered during the time in which parents are already committed to attending the program to maximize parental engagement and minimize burden. Additional research is needed to determine the extent to which these and other parent-focused intervention programs can most effectively support the complex needs of parents, youth and their families seeking treatment for chronic pediatric pain. For example, research that considers the optimal timing and feasibility of delivery of parent-focused support is warranted. Developing options to support parents and youth at the earliest point of treatment for pain symptoms, which typically occurs at the pediatric primary care setting, may provide insight into as of yet untapped potential to prevent further chronicity of symptoms for the pediatric patient and associated caregiving burden for their parent. In addition, research to explore the mechanisms by which these interventions function will help to better understand parents’ regulation of distress to protect adaptive parent-child interactions and to promote positive family dynamics. The lack of mechanistic models is particularly salient in families where consideration is needed about which sociocultural, developmentally appropriate expectations are adaptive and which may need modification to bolster supportive parenting practices—as in the case of families facing pediatric chronic pain. Finally, future studies may investigate the effect of these types of parent intervention programs on parents’ adoption of a new, more flexible, parenting style in the context of parenting a child with chronic pain. 

## 4. Conclusions

This paper summarizes and expands upon a recent symposium presented at the American Psychological Association Annual Convention focusing on parenting a child with chronic pain [1]. An overview of the family as the primary context for care was shared along with a more in-depth consideration of the role that family dynamics and parenting behavior play for families with a child or adolescent with chronic pain. Specific parenting factors, such as parental chronic pain and personal experience of coping with their own chronic pain, parental beliefs about their child’s pain, and parental pain catastrophizing are known to impact caregiving burden for parents of children with chronic pain conditions. Two novel interventions designed to support parents in caring for children with chronic pain conditions in both outpatient (Parents as Coping Coaches) and inpatient (Putting Parents FIRST) settings were described and important aspects of each intervention were reviewed. This study offers initial support that providing intervention to parents of children with chronic pain conditions is feasible and beneficial in both outpatient and inpatient settings.
